# Subthreshold micropulse laser combined with anti-vascular endothelial growth factor therapy for diabetic macular edema: a systematic review and meta-analysis

**DOI:** 10.1007/s00417-024-06460-7

**Published:** 2024-04-25

**Authors:** Hironori Hosoya, Takashi Ueta, Kazunori Hirasawa, Taku Toyama, Tomoyasu Shiraya

**Affiliations:** 1 Department of Ophthalmology, Graduate School of Medicine and Faculty of Medicine, The University of Tokyo, Tokyo, Japan; 2 Department of Ophthalmology, Kitasato University School of Medicine, Tokyo, Japan; 3 Department of Ophthalmology, Showa General Hospital, Tokyo, Japan

**Keywords:** Subthreshold micropulse laser, Vascular endothelial growth factor, Diabetic macular edema, Meta-analysis

## Abstract

**Purpose:**

To evaluate the effects of subthreshold micropulse laser (SML) in addition to anti-vascular endothelial growth factor (VEGF) therapy for diabetic macular edema (DME).

**Methods:**

MEDLINE, EMBASE, and Cochrane Central Register of Controlled Trials were systematically searched for studies that compared anti-VEGF with SML and anti-VEGF monotherapy for DME. Outcome measures were best-corrected visual acuity (BCVA), central macular thickness (CMT), and the number of anti-VEGF injections.

**Results:**

Eight studies including 493 eyes were selected. Four studies were randomized controlled, and the other four were retrospective. Meta-analysis showed that there was no significant difference in BCVA (mean difference [MD] -0.04; 95%CI -0.09 to 0.01 logMAR; P = 0.13;). CMT was thinner in the group of anti-VEGF with SML (MD -11.08; 95%CI -21.04 to -1.12 µm; P = 0.03); however, it was due to a single study that weighed higher, and the sensitivity and subcategory analyses did not support the finding. The number of anti-VEGF injections was significantly decreased in the group of anti-VEGF with SML (MD -2.22; 95%CI -3.02 to -1.42; P < 0.0001).

**Conclusion:**

Current evidence indicates that adding SML to anti-VEGF therapy could significantly reduce the number of anti-VEGF injections compared to anti-VEGF monotherapy, while achieve similar BCVA and CMT.

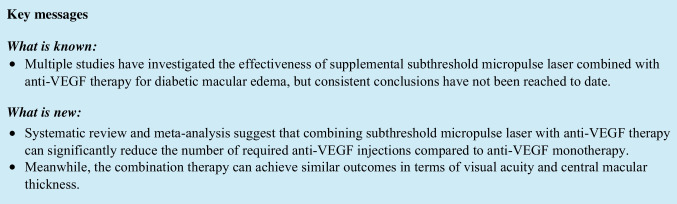

**Supplementary Information:**

The online version contains supplementary material available at 10.1007/s00417-024-06460-7.

## Introduction

Diabetic macular edema (DME) is the leading cause of visual impairment in diabetic patients [1]. Anti-vascular endothelial growth factor (VEGF) therapy has become the standard treatment for DME based on evidence from randomized controlled trials (RCTs) [2]. Alternatively, since the report by the Early Treatment Diabetic Retinopathy Study research group in 1985 demonstrating the efficacy of focal/grid macular photocoagulation in stabilizing visual acuity in patients with DME, this treatment modality has also been widely used [3]. In 2008 Diabetic Retinopathy Clinical Research Network has revealed that focal/grid laser improves visual acuity in patients with DME [4]. However, conventional laser therapy for DME can cause photocoagulation scars through thermal damage to the retinal pigment epithelium (RPE) and the outer retina. While scar formation is considered to provide therapeutic effects by reducing the demand for retinal oxygenation, it can lead to the development of central scotomata and decreased macular sensitivity, which may affect visual function.

The use of subthreshold micropulse laser (SML) was first reported in 1997 as an alternative to conventional laser therapy for retinal diseases [5]. SML delivers short bursts of laser energy with longer intervals between each pulse, allowing the irradiated tissue to cool down and preventing the formation of permanent thermal scars. This technology has been shown to maintain therapeutic effects that are similar to conventional laser, while avoiding the potential side effects associated with scar formation, such as central scotomata and decreased macular sensitivity [6].

Given the established efficacy of anti-VEGF therapy as the primary treatment for DME, it remains an important question whether the addition of SML provides any additional therapeutic benefits. While several studies have investigated this topic, the findings have been inconsistent and limited by small sample sizes. Therefore, the objective of this study is to conduct a comprehensive systematic review and meta-analysis to provide a summary of the current evidence regarding the potential additive effects of SML in combination with anti-VEGF therapy for the treatment of DME.

## Methods

We conducted a systematic review and meta-analysis according to the guidelines set forth in the Cochrane Handbook for Systematic Reviews of Interventions [7]. Our protocol was registered with the International Prospective Register of Systematic Reviews (CRD42022381182) prior to initiating the review.

### Search strategy

Three databases, MEDLINE, EMBASE, and Cochrane Central Register of Controlled Trials were searched from inception to March 2023. Two independent reviewers (H.H. and T.U.) conducted the searches using the following search terms and strategy: (1) *aflibercept.mp.* OR *ranibizumab.mp.* OR *bevacizumab.mp.* OR *anti-VEGF.mp.*, (2) *diabetic macular edema.mp.* OR *diabetic macular oedema.mp.*, (3) *micropulse.mp*. OR *subthreshold.mp.*, and then combined (1) AND (2) AND (3) to identify studies on SML and anti-VEGF therapy for DME. Then the titles and abstracts of the resulting studies were screened, and clearly irrelevant articles were excluded. After this initial screening, full manuscripts of the remaining articles were downloaded and assessed for their eligibility.

### Eligibility criteria

Eligible studies could be randomized or nonrandomized studies reported in English, and had to compare best-corrected visual acuity (BCVA), central macular thickness (CMT), and/or the number of anti-VEGF injections between anti-VEGF monotherapy and anti-VEGF plus SML therapy. The minimum follow-up period was set as 6 months. Noncomparative single-arm studies were not eligible. There was no limitation for years of publication. The process to select eligible studies were undertaken by the tow reviewers (H.H. and T.U.), and consensus regarding eligibility was achieved.

### Data extraction

The extracted information from the included studies included study design, number of patients, age, treatment regimen, types of laser machine, follow-up period, VA and CMT at baseline and last follow-up, the number of injections during the follow-up period. VA were evaluated using logarithm of the minimum angle of resolution (logMAR) values for statistical analysis.

### Risk of bias and certainty of evidence assessment

Risk of bias was assessed using revised Cochrane risk-of-bias tool for randomized trials (RoB2) for randomized studies [8], and risk of bias in non-randomized studies—of interventions (ROBINS-I) for nonrandomized studies [9]. Certainty of evidence for each outcome measures was assessed based on Grading of Recommendations Assessment, Development and Evaluation (GRADE) [10].

### Statistical analysis

EZR version 1.61 (Jichi Medical University, Saitama, Japan) was used for meta-analysis [11]. Mean difference (MD) and 95% confidence interval (95%CI) between the two groups were estimated through common and random effects model. Heterogeneity among the studies was determined by* I*^*2*^. *I*^*2*^ is the proportion of the total variation observed among studies that is not attributed to a sampling error. Heterogeneity is considered high when *I*^*2*^ > 50%. Because* I*^*2*^ values were generally large in the present study, random effects model was considered more suitable to understand the effects of adjuvant SML therapy than common (fixed) effects model. Post-hoc subcategory analyses were performed; excluding nonrandomized studies or studies with higher risk of bias.

## Results

**Fig. 1:**
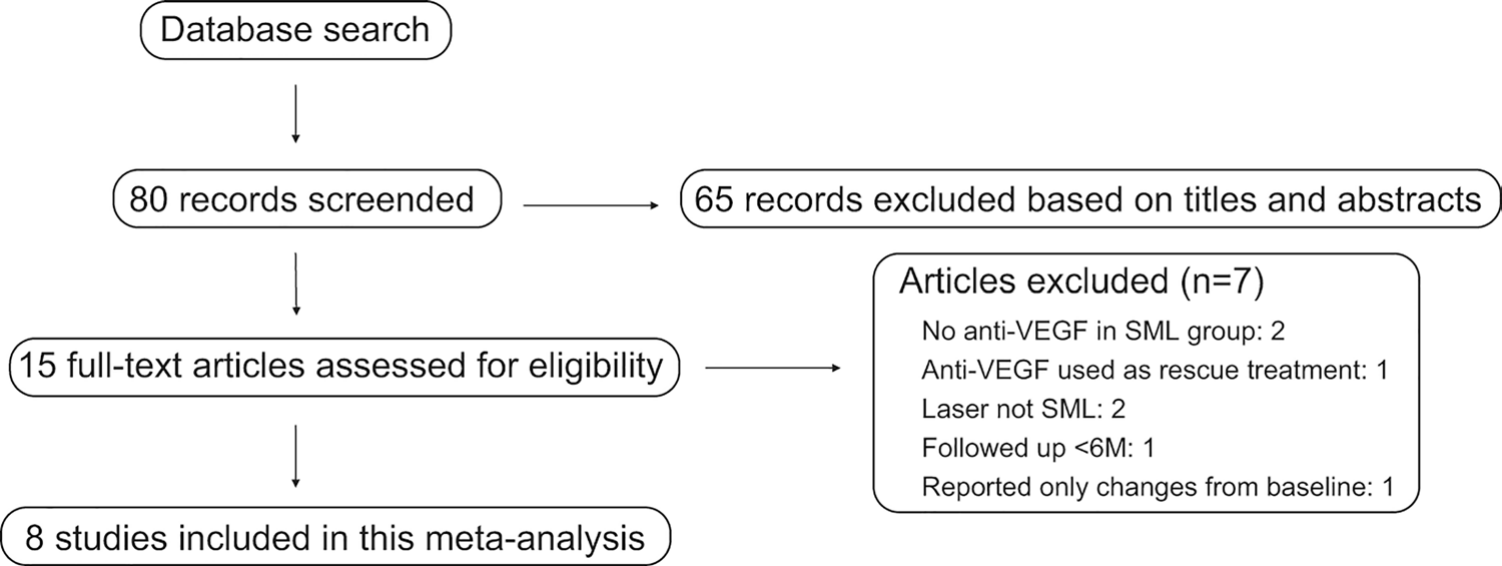
Selection of studies

In the initial screening of the three databases, 80 articles were retrieved, out of which 15 were considered potentially relevant to the study based on the titles and abstracts. Upon detailed assessment of full manuscripts, 7 articles were excluded, leaving 8 studies eligible for meta-analysis (Fig. 1). The excluded articles were excluded due to various reasons such as no anti-VEGF therapy performed in addition to SML (2 study), anti-VEGF therapy performed only as a rescue (1 study), laser was not SML (2 studies), follow-up period was < 6 months (1 study), and only changes from baseline in BCVA and CMT were reported (1 study). The 8 studies [12–19] included in the meta-analysis comprised of 4 RCTs [13–15, 19] and 4 retrospective comparative studies [12, 16–18].

### Characteristics of the included studies

The characteristic of each of the included studies are summarized in Table 1. In total, 493 eyes were included in the meta-analysis: 243 eyes in the anti-VEGF monotherapy group and 250 eyes in the combined anti-VEGF and SML group. The anti-VEGF agent used was bevacizumab in 2 studies, ranibizumab in 2 studies, and aflibercept in 4 studies. The anti-VEGF treatment regimen was three-monthly loading doses followed by pro re nata (PRN) in 6 studies, and PRN protocol from the beginning in 2 studies. SML was performed immediately after the three-monthly anti-VEGF injections in 6 of the included studies [13–18], while it was performed at the timing of the first anti-VEGF injection in 2 studies [12, 19]. The mean number of SML sessions during the follow-up period ranged from 1 to 3.4, and the follow-up period varied from 9 to 20 months across the included studies. In addition, the laser settings in each study were summarized in Table 2. The commonly used laser settings were 577-nm wavelength, 200-µm spot size, 200-ms duration, 5% duty cycle, and around 400mW of energy, which was confluently delivered over the thickened area of the macula including the fovea.

**Table 1:** Characteristics of the included studies

	Country	Design	Treatment	# of SML treatment	Eyes, n	Age, years	HbA1c, %	NPDR, n	Previous PRP	Follow-up Period, mo
Moisseiev, 2018 [12]	USA	Retro	Rani Rani + SML	0 NA	19 19	63.3 ± 8.9 65.3 ± 9.8	NA	10 13	NA	19.1 ± 8.9 23.2 ± 9.0
Khattab, 2019 [13]	Kuwait	RCT	Afli (3 + PRN) Afli (3 + PRN) + SML	0 2.3 ± 0.7	27 27	55.7 ± 3.4 59.4 ± 4.3	NA	20 17	10 7	18 18
Kanar, 2020 [14]	Turkey	RCT	Afli(3 + PRN) Afli(3 + PRN) + SML	0 1.8 ± 0.7	28 28	62.6 ± 9.0 63.4 ± 10.1	8.0 ± 2.4 8.0 ± 2.5	28 28	NA	12 12
Abouhussein, 2020 [15]	Egypt	RCT	Afli (3 + PRN) Afli (3 + PRN) + SML	0 1	20 20	59.5 ± 4.3 60.4 ± 4.2	8.2 ± 1.2 8.7 ± 1.1	20 20	NA	12 12
Altınel, 2021 [16]	Turkey	Retro	Beva(3 + PRN) Beva(3 + PRN) + SML	0 2.1 ± 0.8	40 40	59.8 ± 7.7 60.6 ± 7.2	6.9 ± 0.61 6.9 ± 0.53	40 40	0 within 6 mo	11.1 ± 2.7 11.5 ± 2.0
Matri, 2021 [17]	Tunisia	Retro	Beva(3 + PRN) Beva(3 + PRN) + SML	0 1.41 ± 0.4	49 49	61.3 ± 4.1 67.7 ± 5.2	7.6 ± 0.62 7.7 ± 0.81	49 49	NA	12 12
Bıçak, 2022 [18]	Turkey	Retro	Rani(3 + PRN) Rani(3 + PRN) + SML	0 1	45 52	61.6 ± 6.7 62.4 ± 7.6	6.9 ± 0.59 6.9 ± 0.54	45 52	13 11	9.3 ± 2.3 9.3 ± 2.6
Koushan, 2022 [19]	Canada	RCT	Afli (PRN) Afli (PRN) + SML	0 3.4 ± 1.4	15 15	58.8 ± 9.3 59.8 ± 9.5	NA	NA	NA	12 12

*RCT*, randomized controlled trial; *Retro*, retrospective; *Rani*, ranibizumab; *Afli*, aflibercept; *Beva*, bevacizumab; *SML*, subthreshold micropulse laser; *NPDR*, nonproliferative diabetic retinopathy; *PRP*, panretinal photocoagulation; *mo*, month

**Table 2:** Laser settings in the included studies

	Moisseiev 2018 [12]	Khattab 2019 [13]	Kanar 2020 [14]	Abouhussein 2020 [15]	Altınel 2021 [16]	Matri 2021 [17]	Bıçak 2022 [18]	Koushan, 2022 [19]
Spot size	200 µm	200 µm	160 µm	200 µm	160 µm	200 μm	165 µm	200 μm
Duration	200 ms	200 ms	20 ms	200 ms	200 ms	200 ms	200 ms	20 ms (duration of laser pocket)
Duty cycle	5%	5%	5%	5%	5%	5%	5%	10%
Energy	400mW	400mW	50% of the slightly visible test burn	400mW	50% of the slightly visible test burn	400mW	Half of the slightly visible test burn	90% of the slightly visible test burn
Delivery pattern	150–250 spots in high-density fashion with no overlap to cover the macula	7 × 7 confluent grids over clinically visible thickened retina	Contiguous laser over the area of increased retinal thickness	5 × 5 confluent pattern mode over the entire edematous area including fovea	Confluent laser spots over the area with increased retinal thickness	2 × 2 or 4 × 4 confluent patten mode over the entire edematous area	Grid pattern at central 1000 micron of the macula. If necessary, additional focal laser using confluent laser spots to the areas with increased retinal thickness outside the central 1000 micron	3 × 3 confluent pattern mode over the entire macular area including the foveal center. Non-pattern repeat mode with overlapping burns was also permitted
Wavelength	577 nm	577 nm	577 nm	577 nm	577 nm	577 nm	577 nm	532 nm
Machine	Iridex IQ 577	Iridex IQ 577	Supra 577Y	Iridex IQ 577	Supra 577Y	Iridex IQ 577	Supra 577Y	Supra 532
Lens	Mainster Focal and Grid	Area Centralis	Area Centralis	Mainster Focal and Grid	Area Centralis	Area Centralis	Area Centralis	Not explained

Table 3 displays the baseline BCVA and CMT in each group of the included studies. No statistically significant differences in baseline BCVA and CMT were observed between the two groups in most studies. However, in two studies [12, 17] a difference of approximately 0.1 logMAR and 100 µm was found in mean BCVA and CMT between the two groups, respectively.

**Table 3:** Baseline BCVA and CMT in the included studies

	Treatment group	BCVA at baseline, logMAR	CMT at baseline, µm
Moisseiev, 2018 [12]	Rani	0.41 ± 0.13	408 ± 104
	Rani + SML	0.29 ± 0.12	317 ± 91.5
Khattab, 2019 [13]	Afli(3 + PRN)	1.07 ± 0.19	462 ± 31.2
	Afli(3 + PRN) + SML	1.0 ± 0.2	457 ± 22.6
Kanar, 2020 [14]	Afli(3 + PRN)	0.41 ± 0.11	451 ± 44.9
	Afli(3 + PRN) + SML	0.39 ± 0.09	466 ± 71.8
Abouhussein, 2020 [15]	Afli(3 + PRN)	0.70 ± 0.24	458 ± 82.2
	Afli(3 + PRN) + SML	0.76 ± 0.23	470 ± 78
Altınel, 2021 [16]	Beva(3 + PRN)	0.39 ± 0.23	385 ± 64.1
	Beva(3 + PRN) + SML	0.38 ± 0.21	379 ± 70.3
Matri, 2021 [17]	Beva(3 + PRN)	0.60 ± 0.42	360 ± 22.9
	Beva(3 + PRN) + SML	0.69 ± 0.35	479 ± 14.3
Bıçak, 2022 [18]	Rani(3 + PRN)	0.41 ± 0.25	406 ± 130
	Rani(3 + PRN) + SML	0.43 ± 0.23	427 ± 97
Koushan, 2022 [19]	Afli (PRN)	0.38 ± 0.14	433 ± 104
	Afli (PRN) + SML	0.36 ± 0.21	458 ± 92.8

*Rani*, ranibizumab; *Afli*, aflibercept; *Beva*, bevacizumab; *BCVA*, best corrected visual acuity; *CMT*, central macular thickness; *SML*, subthreshold micropulse laser

### Risk of bias and certainty of evidence

The risk of bias was assessed for each study (Supplemental Table 1 and 2 ). Among the 4 nonrandomized studies, 3 were deemed to have a moderate risk of bias, and the other was considered to have a serious risk of bias. For the 4 randomized studies, 2 were deemed to have a low risk of bias and the other 2 had some risk-of-bias concern. The certainty of evidence assessed using the GRADE approach (Supplemental Table 3), and it was considered low for the evaluations of visual acuity and CMT. However, for the evaluation of the number of anti-VEGF injections, the certainty of evidence was considered moderate after an increase in the certainty of evidence due to the large effect size.

### Meta-analysis on BCVA prognosis

**Fig. 2:**
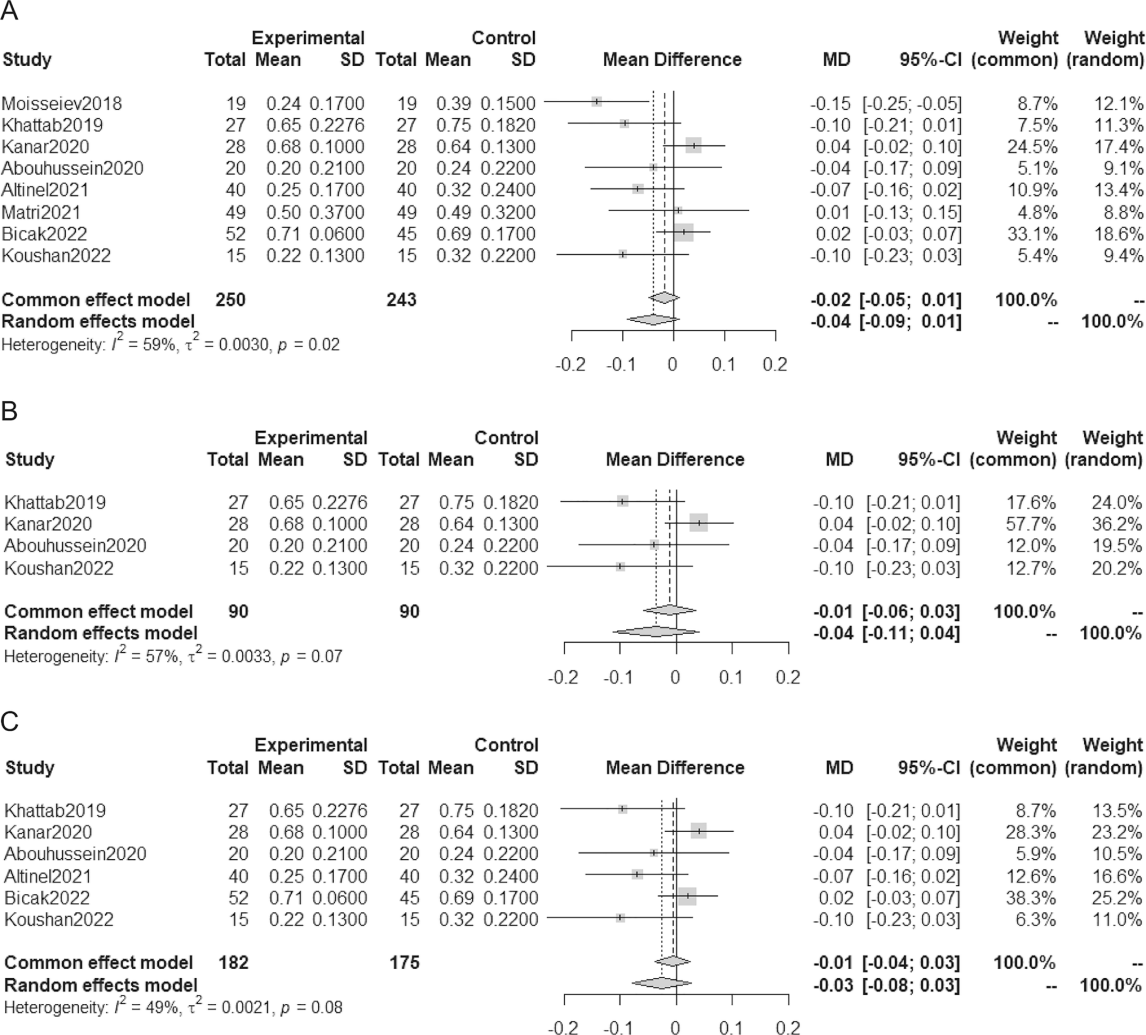
Meta-analysis comparing BCVA after anti-VEGF therapy with SML and anti-VEGF monotherapy; ( A ) meta-analysis including all eligible studies, ( B ) meta-analysis including only randomized studies, and ( C ) meta-analysis excluding studies with a large difference between the two groups in baseline VA and CMT

The meta-analysis included 8 studies with a total of 493 eyes, analyzing the MD in BCVA at one year. Data on the BCVA at the final evaluation of a mean 9.3-month follow-up period were used in one of the studies [18]. The results showed that the addition of SML treatment to anti-VEGF therapy tended to result in better VA, but the difference was not statistically significant (MD -0.04 logMAR; 95%CI -0.09 to 0.01 logMAR; P = 0.13; Fig. 2A). The heterogeneity between trials (*I*^*2*^) was high at 59%.

Further subcategory analyses were performed, including only randomized studies (Fig. 2B) and excluding studies with a relatively large baseline difference in VA and CMT (Fig. 2C). In both subcategory analyses, there was no significant difference in BCVA prognosis between anti-VEGF monotherapy and anti-VEGF with SML.

### Meta-analysis on CMT prognosis

**Fig. 3:**
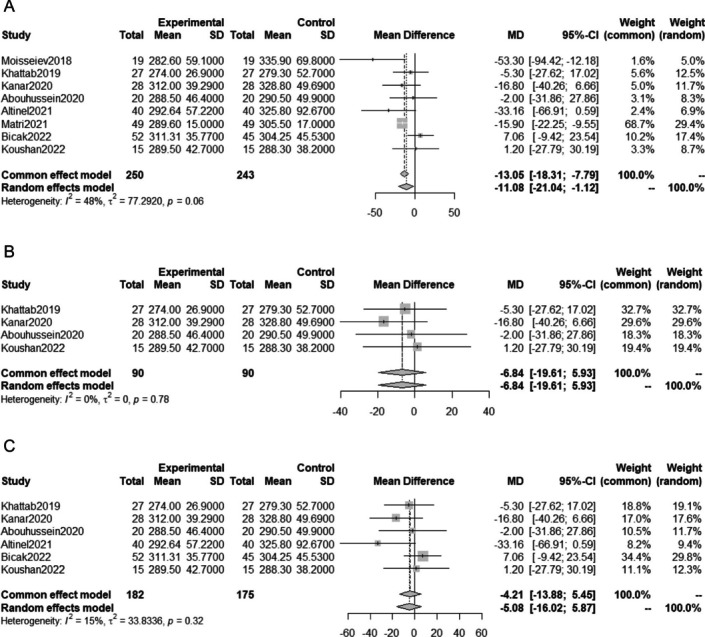
Meta-analysis comparing CMT after anti-VEGF therapy with SML and anti-VEGF monotherapy; ( A ) meta-analysis including all eligible studies, ( B ) meta-analysis including only randomized studies, and ( C ) meta-analysis excluding studies with a large difference between the two groups in baseline VA and CMT

The analysis of MD in CMT approximately one year after treatment was conducted using data from eight studies including 493 eyes. Data on the CMT at the final evaluation of a mean 9.3-month follow-up period were used in one of the studies [18]. The results of the meta-analysis using a random effects model indicated that SML treatment in addition to anti-VEGF therapy may significantly reduce CMT (MD -11.08 µm; 95%CI -21.04 to -1.12 µm; P = 0.03; Fig. 3A). However, one study had a higher weight due to a smaller standard deviation, and a sensitivity analysis was performed excluding this study, which showed no statistical significance between the two groups (MD -9.81 µm; 95%CI -16.20 to 2.63 µm; P = 0.14; Supplemental Fig. 1).

Subcategory analyses were performed, including only randomized studies (Fig. 3B) and excluding studies with relatively large baseline differences in VA and CMT (Fig. 3C). In both subcategory analyses, there was no significant difference in CMT between anti-VEGF monotherapy and anti-VEGF with SML.

### Meta-analysis on the number of anti-VEGF injections

**Fig. 4:**
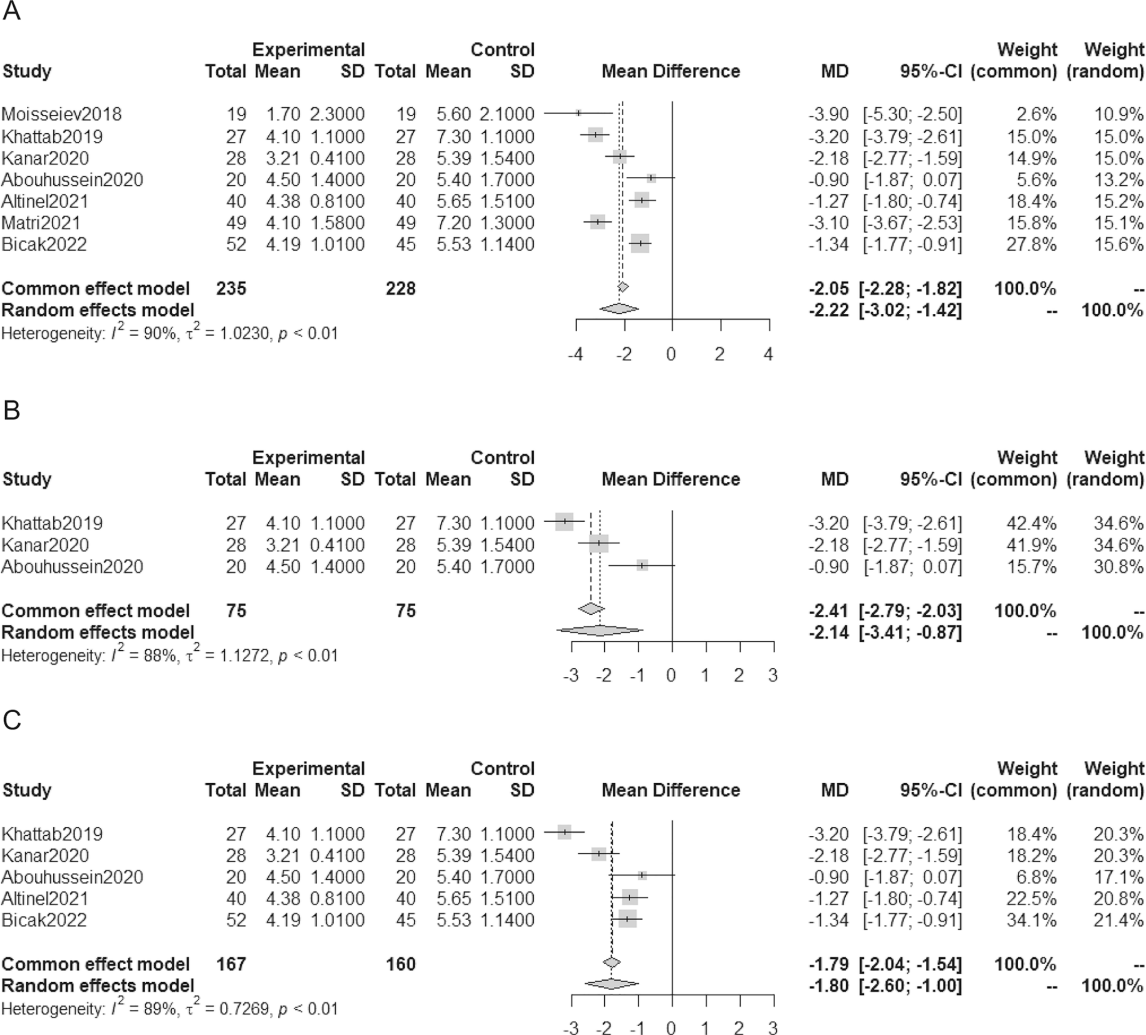
Meta-analysis comparing the number of anti-VEGF injections after anti-VEGF therapy with SML and anti-VEGF monotherapy; (**A**) meta-analysis including all eligible studies, (**B**) meta-analysis including only randomized studies, and (**C**) meta-analysis excluding studies with a large difference between the two groups in baseline VA and CMT

Similarly, MD in the number of anti-VEGF injections during one year was analyzed using 7 studies with 463 eyes. Data from one of the studies included the number of injections administered during an 18-month follow-up period [13], while the other study reported the number of injections given during a mean of 9.3 months follow-up period [18]. The meta-analysis showed that SML treatment in addition to anti-VEGF therapy significantly reduced the number of injections required (MD -2.22; 95%CI -3.02 to -1.42; P < 0.0001; Fig. 4A).

Subcategory analyses performed including randomized studies only (Fig. 4B), and excluding studies with relatively large baseline difference in VA and CMT (Fig. 4C). Both subcategory analyses showed a significant decrease in the number of injections in the anti-VEGF with SML group compared with the anti-VEGF monotherapy group.

## Discussion

Anti-VEGF therapy has been the first choice to treat center-involving DME [2], but the need for frequent injections can be a burden for both patients and clinicians. To address this issue, combination therapies with macular laser treatments, including focal/grid [20–22], navigated [23, 24], and SML, have been proposed and are being used in ophthalmic practice. Our meta-analysis suggests that SML in combination with anti-VEGF therapy can reduce the number of intravitreal injections required, while maintaining efficacy in improving BCVA and CMT.

Previously, combination therapy of macular grid/direct photocoagulation with anti-VEGF therapy had been attempted, but its effectiveness has not yet been established [24, 25]. Furthermore, conventional macular photocoagulation can cause irreversible retinal damage and lead to atrophic creep, potentially resulting in scotomata and decreased visual function. In 1997, Friberg, et al. reported the use of SML for DME using 810 nm diode laser [5]. 577-nm wavelength micropulse laser became available later, and it is not absorbed by the xanthophyll pigment in the macula and is more absorbed by melanin in the retinal pigment epithelium than the 810 nm wavelength [15, 26]. It has been expected to have similar or better therapeutic effects than conventional laser in stabilizing or improving BCVA and CMT in patients with DME without causing laser scar or irreversible visual complications [6, 27, 28]. Recently, DIAMOND trial has shown that patients treated with SML require fewer rescue anti-VEGF injections compared to those treated with conventional macular laser, while achieving non-inferiority and equivalence in BCVA and CMT outcomes [6].

The present meta-analysis shows a consistent trend of a smaller number of anti-VEGF injections being necessary when SML is used as an adjuvant therapy. This significant decrease in the number of anti-VEGF injections may be attributed to several conditions. Firstly, in 6 out of the 8 studies included in this meta-analysis, SML was performed after 3 monthly loading doses of anti-VEGF therapy. This implies that SML was performed after a temporary decrease in CMT. A previous study has reported that SML is more efficacious in treating milder residual DME of CMT < 300 µm after anti-VEGF therapy [29]. Although the exact mechanisms are unclear, it is hypothesized that the concentration of beneficial cytokines produced by SML may be diluted in cases of severe DME [29]. Additionally, it is possible that sufficient SML energy may not reach the retinal pigment epithelium due to the thickness of the edematous macula, resulting in insufficient production of these cytokines.

Secondly, all of the included studies used a PRN regimen for anti-VEGF therapy, either from the beginning or after three monthly loading doses (3 + PRN). This PRN protocol may have made it easier to observe differences in the number of injections because injections were only performed when predefined criteria for worsening BCVA and/or CMT were met. This is in contrast to the treat-and-extend regimen, in which injections are given even when BCVA and CMT are stable, making it difficult to detect differences in the number of injections between the treatment groups. Previous studies evaluating the effects of navigated macular laser in addition to anti-VEGF therapy have also observed a significant decrease in the number of injections under PRN protocols [23], but not under treat-and-extend protocols [24].

The present meta-analysis indicates that the addition of SML therapy does not result in a significant change in BCVA and CMT in patients with DME. The meta-analysis on CMT showed a significant reduction with the additional SML in the primary analysis, which was mainly driven by one study that had a substantial weight of about 30% even in the random effects model. However, sensitivity analysis excluding this study or conducting subcategory analyses did not show a significant difference in CMT outcome between the two groups.

There are several limitations to this study. Firstly, half of the included studies were retrospective in nature, which may have introduced bias and limitations in the quality of data. Since in some studies there were a considerable difference in baseline VA and CMT, we carefully conducted sensitivity analyses that excluded such studies, and tested the robustness of the conclusions. Secondly, while the other half of the studies were RCTs, the sample size in each study was relatively small. This may have affected the precision of the estimates and contributed to the high heterogeneity observed across studies. Thirdly, the variations in the baseline severity of DME and DR, the types of anti-VEGF agents used, and the number of SML treatments administered may have also contributed to the high heterogeneity observed across the studies. Fourthly, due to the retrospective nature of some of the studies, the certainty of evidence assessed by GRADE was considered to be "low" for BCVA and CMT outcomes. Finally, while the certainty of evidence for the effect of SML on the number of anti-VEGF injections was considered to be "moderate" due to the significant difference observed between the two groups, further studies are needed to confirm the findings and evaluate the long-term outcomes of SML as an adjuvant therapy for DME.

In conclusion, this systematic review and meta-analysis provide evidence for the potential benefit of SML as an adjunct therapy to reduce the number of anti-VEGF injections while maintaining similar BCVA and CMT outcomes compared to anti-VEGF monotherapy. However, due to the limitations of the included studies, further large-scale RCTs are needed to confirm these findings and determine the optimal treatment regimen for SML in combination with anti-VEGF therapy.

## Supplementary Information

Below is the link to the electronic supplementary material.

Supplementary file1 (PDF 166 KB)
