# Supplementary material for: Subthreshold micropulse laser combined with anti-vascular endothelial growth factor therapy for diabetic macular edema: a systematic review and meta-analysis
Source: Graefes Arch Clin Exp Ophthalmol. 2024 Apr 25;262(10):3073–83. doi: 10.1007/s00417-024-06460-7 (PMC11458775; doi:10.1007/s00417-024-06460-7)

Subthreshold micropulse laser combined with anti-vascular endothelial growth factor therapy for diabetic macular edema: A systematic review and meta-analysis

Hironori Hosoya, MD[1]; Takashi Ueta, MD, PhD[1]; Kazunori Hirasawa, PhD;[2] Taku Toyama, MD, PhD;[1] Tomoyasu Shiraya, MD, PhD[1,3]

[1]Department of Ophthalmology, Graduate School of Medicine and Faculty of Medicine, The University of Tokyo

[2]Department of Ophthalmology, Kitasato University School of Medicine, Tokyo Japan

[3]Department of Ophthalmology, Showa General Hospital, Tokyo, Japan

Correspondence:

Takashi Ueta, MD, PhD

Email: ueta-tky@umin.ac.jp

Supplemental Table 1. Risk of bias assessment; ROBINS-I for nonrandomized studies

	Pre-intervention		At intervention	Post-intervention				Overall risk of bias
	Confounding	Selection bias	Misclassification	Deviations from intended interventions	Missing data	Measurement errors	Selective reporting	
Moisseiev, 2018	moderate	serious	moderate	low	low	low	low	serious
Altınel, 2021	moderate	moderate	moderate	low	moderate	low	low	moderate
Bıçak, 2022	moderate	moderate	moderate	low	low	low	low	moderate
Matri, 2021	moderate	moderate	moderate	low	low	low	low	moderate

Supplemental Table 2. Risk of bias assessment; RoB2 for randomized studies.

	Bias arising from randomization process	Bias due to deviations from the intended interventions	Bias due to missing outcome data	Bias in measurement of the outcome	Bias in selection of the reported result	Overall risk of bias
Khattab, 2019	Some concern	Low risk	Low risk	Low risk	Low risk	Some concern
Kanar, 2020	Low risk	Low risk	Low risk	Low risk	Low risk	Low risk
Abouhussein, 2020	Some concern	Low risk	Low risk	Low risk	Low risk	Some concern
Koushan, 2022	Low risk	Low risk	Low risk	Low risk	Low risk	Low risk

Supplemental table 3. Certainty of evidence assessment based on GRADE.

	Initial certainty of evidence	Risk of bias	Inconsistency	Indirectness	Imprecision	Publication bias	Dose-response gradient	Large effect size	Effet of plausible residual confounding	Final certainty of evidence
Visual acuity at 1 year	Low	±0	±0	±0	±0	±0	±0	±0	±0	Low
CRT at 1 year	Low	±0	±0	±0	±0	±0	±0	±0	±0	Low
# of injections	Low	±0	±0	±0	±0	±0	±0	+1	±0	Moderate

Supplemental Figure 1. A sensitivity meta-analysis on CRT that excludes a study of a highest weight.

Study	Experimental Total	Experimental Mean	Experimental SD	Control Total	Control Mean	Control SD	Mean Difference	MD	95%-CI	Weight (common)	Weight (random)
Moisseiev2018	19	282.60	59.1000	19	335.90	69.8000		-53.30	[-94.42; -12.18]	5.2%	7.8%
Khattab2019	27	274.00	26.9000	27	279.30	52.7000		-5.30	[-27.62; 17.02]	17.8%	17.4%
[illegible]	[illegible]	[illegible]	[illegible]	[illegible]	[illegible]	[illegible]		[illegible]	[illegible]	[illegible]	[illegible]

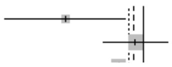

Supplement: Supplementary file 1 — Supplementary file1 (PDF 166 KB) [file 417_2024_6460_MOESM1_ESM.pdf]
